# Studies on Genomic DNA Stability in Aluminium-Maltolate Treated Aged New Zealand Rabbit: Relevance to the Alzheimers Animal Model

**DOI:** 10.4021/jocmr2009.09.1265

**Published:** 2009-10-16

**Authors:** Obulesu Magisetty, Dowlathabad Muralidhara Rao, Shama Sundar N. M

**Affiliations:** a Department of Anatomy, JSS Medical College, Mysore, India; b Department of Biotechnology, Sri Krishnadevaraya University, Anantapur, India

## Abstract

**Background:**

Alzheimers disease (AD) is a devastative neurodegenerative disorder. Lack of substantial animal model that can unravel molecular underpinnings has been a major lacuna which limited the understanding of the etiology of the disease in turn limiting the employment of potential therapeutic strategies to combat the disease for a few decades. Our studies for the first time provided substantial animal model and tattered the etiology of the disease at a molecular level.

**Methods:**

In this study DNA was isolated from Hippocampus (H), Midbrain (M) and Frontal Cortex (Fc) of control and aluminium maltolate (Al-M) treated aged New Zealand rabbit brain. DNA damage has been studied using Agarose gel electrophoresis, Ethidium Bromide (EtBr) binding and Melting temperature techniques.

**Results:**

Al-M treated aged New Zealand rabbit's H and M showed higher DNA damage compared to corresponding controls, where as Fc showed mild DNA damage compared to corresponding controls.

**Conclusions:**

This study tangibly provides substantial molecular level understanding of the disease in turn providing an adequate platform to streamline potential therapeutic strategies.

**Keywords:**

Alzheimer’s disease; Aluminium maltolate; Animal model; DNA damage

## Introduction

Although adequate research has been progressing for a century, the understanding of the etiology of Alzheimers disease (AD) has not been completely unraveled due to the lack of substantial animal model [1,2]. The biochemical events entailed in the neuronal cell loss are not clear till date [1,3]. The majority of the animal models worked till date could demonstrate single event expression such as extra cellular Aβ deposition, intraneuronal neurofilamentous aggregation of proteins similar to neurofibrillary tangles, oxidative stress and apoptosis [1,4]. However, the intracisternal injection of aluminium maltolate (Al-M) into aged New Zealand rabbit replicates the neuropathological, biochemical and behavioural changes found in AD [1, 5-13]. Lovell et al reported DNA damage like single strand/double strand breaks, base specific oxidation like G* specific oxidation [14-19]. Increased DNA oxidation has also been noticed in AD [20-22]. A few studies have shown that DNA repair failure is a prominent feature in AD [23,24]. The deterioration of the DNA repair system takes place with aging. Abnormal cell cycle regulation and/or accumulated DNA damage also leads to AD [25]. A few animal models were developed to study the genes responsible for this disease and the increase of specific transcripts [26]. The pathological events like hyperphosphorylation of tau, formation of neurofibrillary tangles, Aβ deposition, were studied [27-29]. Introduction of Aβ 1-42 into rabbits also provided animal model for AD [30].

Accumulation of DNA damage may also lead to AD [31]. Limited work has been done on DNA stability in this animal model so far. The present work has been carried out to unravel the molecular underpinnings of the disease pathology.

## Materials and Methods

Maltol, Al (NO3)3. 9 H_2_O, Agarose, Ethidium Bromide (EtBr), Hepes, Tris, Hepes and Tris buffers were purchased from Sigma Chemicals (USA). All other chemicals were of analytical grade and were purchased from Sisco Research Labs, Mumbai, India.

### Al-M preparation

Al-M was prepared from maltol (3-hydroxy-2methyl-4H pyran-4-one) following the method of Finneagan [32]. Maltol and Al (NO_3_)_3_. 9 H_2_O were mixed in 3:1 ratio. The pH was adjusted to 8.6 and heated for a few minutes. This aluminium-maltolate was used in the present study to understand the Aluminium's effect on genomic DNA. Aluminium complex is hydrolytically stable from pH 2.0 to 12.0. This complex enhances free Aluminium existence by 60-70% at neutral pH compared to any other inorganic or organic Aluminium complex.

Aluminium speciation chemistry is a complex phenomenon, hence to overcome this observation Aluminium-maltolate (Al-M) was used in the present investigation.

### Animal treatment and tissue processing

All the animals were maintained in JSS animal house in single stainless steel cages. The experiments were done according to the institutes ethical committee and INSA guidelines. Aged Rabbits (3.5 to 4 yrs) from JSS Medical College Animal Colony were used. Six Rabbits were treated with Al-M intracisternally, while 6 rabbits with 0.9% saline injection were used as control. The intracisternal injection of Al-M into aged New Zealand rabbits was done as described by Savory [11,33]. The animals were decapitated and their brains were quickly removed, snap-frozen in N-methylbutane at a temperature of -40 ^o^C with liquid nitrogen and stored at -80 ^o^C [34]. The natomical localization of hippocampus, midbrain and cortical regions were accomplished using the designation outlined in an atlas of the rabbit brain and spinal cord [35].

### Isolation of DNA from brain tissue

Genomic DNA from control and Al-M treated brain tissues were isolated by phenol-chloroform extraction protocol [36]. Tissue pieces were transferred into an autoclaved porcelain mortar and pestle (all glass wares, mortar, pestle were autoclaved before using them in order to avoid bacterial contamination). Liquid nitrogen was poured into the mortar and the tissue was frozen. Tissue was ground thoroughly with pestle with frequent additions of liquid nitrogen. Sufficient quantity of liquid nitrogen was poured into the mortar and swirled. Tissue homogenate was transferred into a sterile tube and the liquid nitrogen was allowed to evaporate. A sterile spatula was used to transfer the powdered tissue into a graduated tube. Lysis buffer 50 mM Tris-Hcl (pH 8.0), 10 mM EDTA, and 100 mM NaCl was added into the tube along with 15 ug per ml of proteinase K and 2% SDS final volume. One ml of lysis buffer was used for every 500 mg of tissue. Lysis buffer was pre warmed, added proteinase K after first 2h. The homogenate was incubated at 37 ^o^C in a water bath for 12-16 h or over night. After the completion of incubation, the incubated lysate was transferred to an autoclaved 50 ml conical flask and equal volume of tris-saturated phenol (pH 8.0) was added and mixed thoroughly, either manually or mechanically for 10 min. The lysate was centrifuged for 10 min at 10,000 rpm at 13 ^o^C. The supernatant was collected into a fresh autoclaved 50 ml conical flasks and half volume of tris saturated phenol and chloroform: isoamyl alcohol was added and mixed thoroughly. One part phenol: 1 part chloroform (C) and isoamylalcohol (IA) mixture (C: IA= 23:1). Tris-saturated phenol was stored in amber colored bottles at low temperature to avoid oxidation of phenol. The supernatant and tris-saturated phenol-chloroform mixture was centrifuged at 5000 rpm at 4 ^o^C.

The upper aqueous layer was collected into a fresh tube and 1/3 volume of sodium acetate (pH 5.5) and equal volume of chilled absolute ethanol was added. DNA was precipitated by slowly swirling the tube manually. DNA was washed twice with 70% alcohol and once with absolute alcohol to remove excess salt and vacuum dried. The vacuum dried DNA was dissolved in 1 ml of TE buffer (10 mM Tris-Hcl 1mM EDTA, pH 8.0). The DNA isolated from cells contains RNA also which was removed by digesting the preparation with RNAse enzyme. RNAse solution was kept in boiling water for 10 min so as to inactivate any DNAse because the RNAse may contain DNAse also. RNAse can also be added before Proteinase K treatment, and incubated at 37 oC for 1 hour then start Proteinase K treatment (Add 1 g of RNAse/ml of lysis buffer for 30 min).

### DNA concentration and purity

DNA was quantified by recording its optical density at 260 nm by UV spectrophotometer. Absorbance of isolated DNA was read at 280 nm. A260/A280 nm ratio was measured [36].

### Neutral agarose gel electrophoresis

The genomic DNA integrity and damage was assessed by running neutral gel electrophoresis. The migration pattern in neutral gels reflects the double strand breaks present in the DNA. Neutral gels were electrophoresed on 1.5% agarose gels in Tris-acetate EDTA buffer (pH 8.0) at 4V/cm for 4h. Three ug of DNA was loaded in each well [36].

### EtBr binding to DNA and Scatchard plots

The binding of EtBr to control and Al-M treated aged rabbits brain DNA was measured in 0.01 M HEPES buffer (pH 7.0) using Spectrofluorimeter (HITACHI, Japan) by taking 1:1 (w/w) DNA/EtBr before measuring fluorescence emission. DNA/EtBr solutions were excited at 535 nm, and emission intensity was monitored at 600 nm using HITACHI F-2000 Fluorescence Spectrophotometer. The amount of EtBr bound to DNA was calculated using the independent binding equation of Scatchard [36,37].

### Melting temperature profiles

The melting curves for control and Al-M treated rabbits brain DNA was carried out in 0.01 M HEPES buffer (pH 7.4) by recording UV absorbance at 260 nm at different temperatures 1 oC /min using a Gilford Response II UV spectrophotometer fitted with thermostat control. The temperature range was between 25-95 ^o^C. Tm values were determined graphically from the absorbance versus temperature plots. The temperature point at which there is a 50% absorbance shift was taken as melting temperature (Tm) of the DNA sample. The precision of Tm values was estimated [36].

## Results

### Behavioural features

Al-M treated aged New Zealand rabbits showed following behavioural changes like reduced food intake, isolation behavior, forward head tilting, increased eye blinking, and hemiplegia.

### Agarose Gel electrophoresis

**Figure 1:**
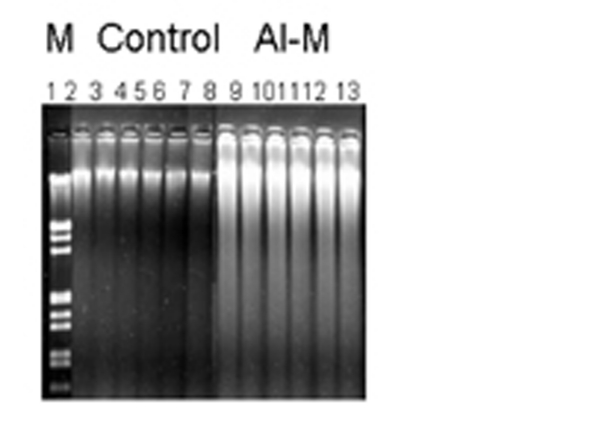
Indicates migration pattern of control and Al-M treated H DNA. Left lane M is Lambda DNA / EcoRI/HindIII double digest marker, 2-7 are control samples of H showing intact DNA where as 8-13 showed diffused bands indicating DNA damage.

**Figure 2:**
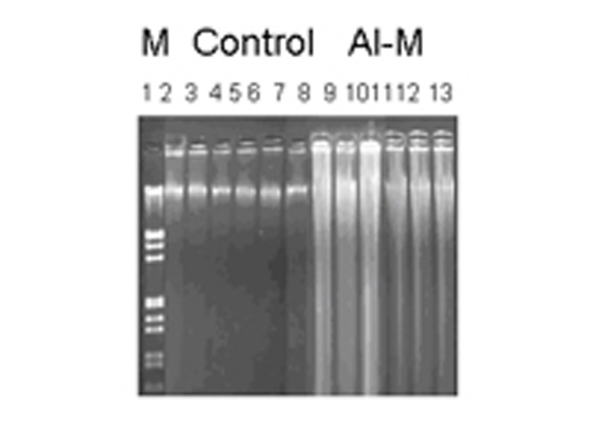
Indicating migration pattern of control and Al-M treated M DNA. Left lane M is Lambda DNA / EcoRI/HindIII double digest marker, 2-7 are control samples of M showing intact DNA where as 8-13 showed diffused bands indicating DNA damage.

**Figure 3:**
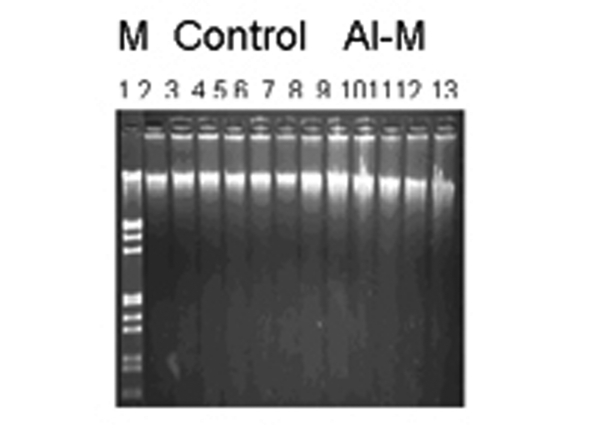
Indicating migration pattern of control and Al-M treated Fc DNA. Left lane M is Lambda DNA / EcoRI/HindIII double digest marker, 2-7 are control samples of Fc showing intact DNA, 8-13 are Al-M treated DNA.

DNA damage was observed by neutral agarose gel electrophoresis. Figure 1 shows neutral gels of genomic DNA from H of control and Al-M treated aged New Zealand white rabbits. Figure 2 shows neutral gels of genomic DNA from M of control and Al-M treated aged New Zealand white rabbits. Figure 3 shows neutral gels of genomic DNA from Fc of control and Al-M treated aged New Zealand white rabbits.

It has been observed that H demonstrated intense diffusion of the DNA bands indicating highest damage. The next highest damage has been observed in M. Frontal Cortex showed mild damage compared to other brain regions.

### Melting temperature profile

The values mentioned in Table 1 are the temperatures at which DNA is half denatured. The values are average of triplicates. The data in Table 1 showed that the Tm is low for Al-M treated H and M compared to the corresponding controls indicating considerable DNA damage or reduced stability. There was a little difference in the Tm of Fc indicating mild damage.

**Table 1:** Melting Temperature (Tm) of DNA isolated from control and Al-M treated aged rabbits

Sample	Control	Al-M treated
Hippocampus	86.8 ± 0.2	74 ± 0.3
Mid Brain	89.7 ± 0.7	85.1 ± 0.1
Frontal Cortex	85.1 ± 0.1	82.7 ± 0.7

Values mentioned are as degree centigrade with means ± SD. The point of 50% hyperchromic shift was calculated as Tm. Al-M treated H and M showed considerable reduction in Tm compared to corresponding controls.

### EtBr binding studies

**Figure 4:**
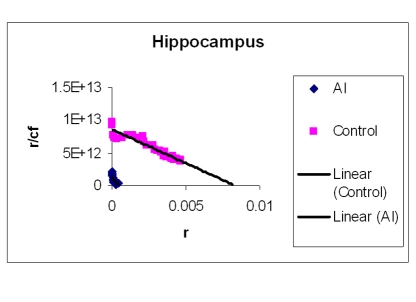
Showing Scatchard plot of ethidium bromide binding to DNA isolated from H. EtBr concentrations were increased to a standard concentration of DNA in a 1 ml reaction mixture. Fluorescence was obtained by keeping the excitation at 535 nm and emission at 600 nm.

The amount of EtBr molecules bound per base pair of DNA is represented in Table 2. Two representative Scatchard plots of control and treated samples were drawn (Fig. 4, 5 , 6) for H, M and Fc respectively). The values in Table 2 are average of triplicates. Table 2 shows that Al-M treated H bound less EtBr compared to corresponding control indicating that DNA in this region is not intact. The next highest difference in EtBr binding has been observed in M. Mild difference in EtBr binding was observed in Fc.

**Table 2:** EtBr binding assay of of DNA isolated from control and Al-M treated aged rabbits

Sample	Control	Al-M treated
Hippocampus	0.004 ± 0.0003	0.0008 ± 0.0001
Mid Brain	0.003 ± 0.0002	0.001 ± 0.0002
Frontal Cortex	0.0035 ± 0.0005	0.002 ± 0.0001

EtBr bound per base pair of DNA from control and Al-M treated H, M & Fc were calculated using Scatchard plots. Values given are as mean of number of EtBr molecules bound per base pair of DNA ± SD

**Figure 5:**
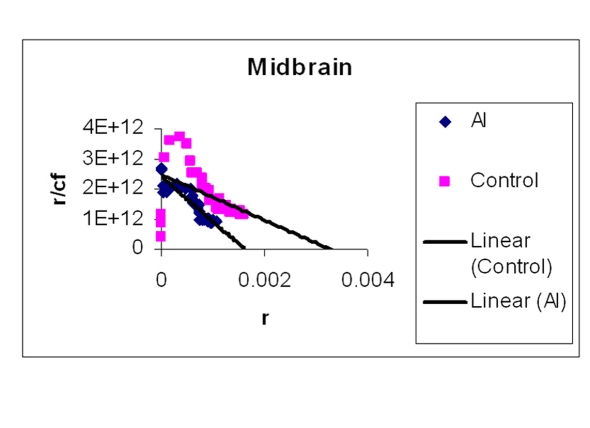
Showing Scatchard plot of ethidium bromide binding to DNA isolated from M. EtBr concentrations were increased to a standard concentration of DNA in a 1 ml reaction mixture. Fluorescence was obtained by keeping the excitation at 535 nm and emission at 600 nm.

**Figure 6:**
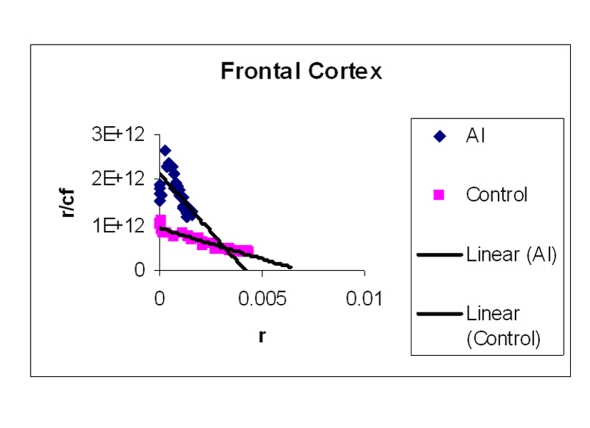
Showing Scatchard plot of ethidium bromide binding to DNA isolated from Fc. EtBr concentrations were increased to a standard concentration of DNA in a 1 ml reaction mixture. Fluorescence was obtained by keeping the excitation at 535 nm and emission at 600 nm.

### Melting temperature

**Figure 7:**
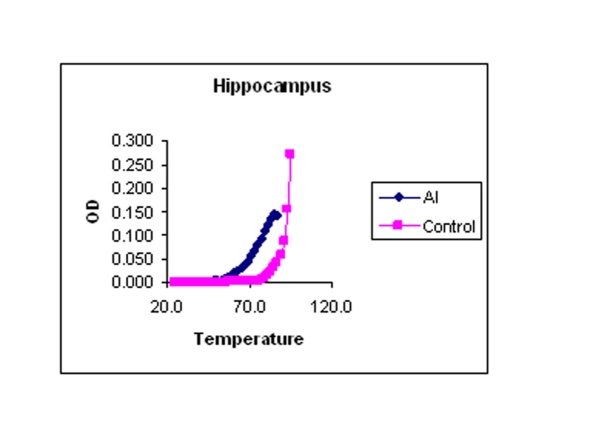
Indicating melting temperature profile of H DNA isolated from control and Al-M treated rabbit brains.

**Figure 8:**
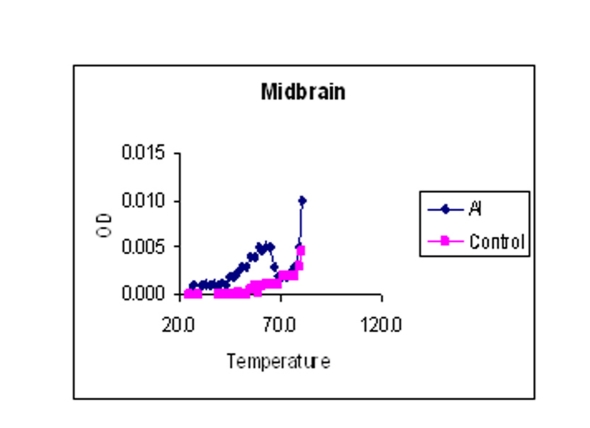
Indicating melting temperature profile of M DNA isolated from control and Al-M treated rabbit brains.

**Figure 9:**
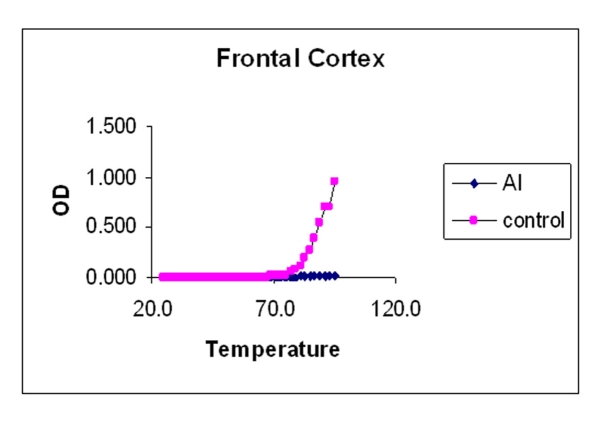
Indicating melting temperature profile of Fc DNA isolated from control and Al-M treated rabbit brains.

The melting temperature profiles of H, M, and Fc DNA isolated from control and Al-M treated rabbit brains were shown in Figure 7, 8, 9, respectively.

## Discussion

Genomic DNA integrity plays pivotal role in the survival of an organism. The damage caused to its integrity shows deleterious effects on the health eventually leading to death. DNA repair capacity reduces with advancing age thus leading to the accumulation of the DNA damage. Oxidation of bases, strand breaks, formation of adducts, are the considerable factors in AD pathogenesis [15]. The animals tested so far could not provide substantial information on the etiology of the disease.

The animal models studied till date could demonstrate single pathological feature but failed to reciprocate the complete pathology of the AD. This lacuna made the insight into the pathology of the disease an uphill task for a few decades. Al is known to induce neurodegeneratiion although its role has been suspicious for a few decades. Since the advent of neurotoxicological studies many Al salts like AlCl3 [38] have been employed which produced insoluble complexes at neutral pH. Al-M has been chosen to be a neurotoxic agent for our studies, since maltolate aggravates the neurotoxicity of the Al. Al-M induced rabbits mimic AD pathology. Al-M has following properties which made it an appropriate neurotoxin: 1, very high metal solubility at pH 7.0; 2, prominent kinetic restrictions to ligand exchange reactions in neutral solution [1]. This work accentuates the role of Al in neurodegeneration.

Our studies showed DNA damage in Al-M treated aged rabbits. Gel pattern showed the damage which is substantiated by Tm and EtBr values. Low Tm and less binding of EtBr to the DNA emphasize the DNA damage [36].

Although adequate research has been going on for almost a century, the etiology of the AD has not yet been unraveled completely. The above observations emphasize the fact that there is severe damage in H which leads to the memory loss. The next highest damage has been noticed in M. The damage observed in Fc is considerably less. These findings are in accordance with the AD pathology. DNA damage or stability reduction plays pivotal role in AD. This work throws light on molecular understanding of pathology and tangibly gives substantial information about the etiology of the disease which stands as a suitable corner stone to streamline the potential therapeutic strategies.
